# Lack of artemisinin resistance in *Plasmodium falciparum* in northwest Benin after 10 years of use of artemisinin-based combination therapy

**DOI:** 10.1051/parasite/2016028

**Published:** 2016-07-21

**Authors:** Aurore Ogouyèmi-Hounto, Georgia Damien, Awa Bineta Deme, Nicaise T. Ndam, Constance Assohou, Didier Tchonlin, Atika Mama, Virgile Olivier Hounkpe, Jules Doumitou Moutouama, Franck Remoué, Daouda Ndiaye, Dorothée Kinde Gazard

**Affiliations:** 1 Unité d’Enseignement et de Recherche en Parasitologie – Mycologie/Faculté des Sciences de la Santé; 01 BP 188 Cotonou Bénin; 2 Laboratoire du centre de lutte intégrée contre le paludisme; 01 BP 188 Cotonou Bénin; 3 Institut de Recherche pour le Développement. UMR 224-MIVEGEC; 08 BP 841 Cotonou Bénin; 4 Laboratory of Parasitology Mycology, Aristide le Dantec Hospital BP 16477 Dakar Senegal; 5 UMR 216 MERIT-IRD Parasitology Department Noguchi Memorial Institute for Medical Research College of Health Sciences, University of Ghana P.O. Box LG581 Legon Accra Ghana; 6 Centre de santé de la Commune de Djougou 02 BP 681 Porto Novo Benin; 7 Centre de santé de la Commune de Cobly BP 40 Tanguiéta Bénin

**Keywords:** *Plasmodium falciparum*, Malaria, Resistance, Artemisinin, *K13* propeller, Benin

## Abstract

**Aim**: In Benin, artemisinin-based combination therapy (ACT) has been recommended as the first-line treatment for uncomplicated *Plasmodium falciparum* malaria since 2004. The emergence in Southeast Asia of parasites that are resistant to artemisinins poses a serious threat to global control of this disease. The presence of artemisinin resistance genotypes in parasite populations in Benin is currently unknown. The present study investigated the prevalence of relevant *K13-propeller* gene polymorphisms in parasite isolates from the north-western region of Benin.

**Method**: *Plasmodium falciparum* isolates were collected from children with a confirmed diagnosis of malaria aged 6 months to 5 years in two towns, Cobly and Djougou, in the north-western part of Benin. The study was conducted during the rainy season from July to November 2014 in local health facilities. The *K13-propeller* gene was amplified in parasite isolates using nested PCR and subsequently sequenced.

**Results**: A total of 108 children were recruited into the study. The efficiency of amplification reactions was 72% (78/108). The propeller domain of the *K13* gene was successfully sequenced in 78 *P. falciparum* isolates; all of them were wild type with no polymorphisms detectable.

**Conclusion**: The absence of mutations in the *K13* gene indicates that *P. falciparum* parasite populations in the study area are still fully susceptible to artemisinins.

## Abstract (fr)

**Contexte :** Au Bénin, depuis 2004, les combinaisons thérapeutiques à base d’artémisinine ont été recommandées comme traitement de première intention du paludisme à *Plasmodium falciparum* non compliqué. L’émergence en Asie du Sud-Est de parasites qui sont résistants à l’artémisinine pose une menace sérieuse pour la lutte mondiale contre cette maladie. La présence de génotypes de résistance à l’artémisinine dans les populations de parasites au Bénin est actuellement inconnue. Cette étude a étudié la prévalence des polymorphismes du gène *K13-propeller* chez des parasites isolés de la région nord-ouest du Bénin.

**Méthodes :** Les isolats de *Plasmodium falciparum* ont été recueillis auprès d’enfants ayant un diagnostic confirmé de paludisme, âgés de 6 mois à 5 ans, dans deux villes, Cobly et Djougou, dans la partie nord-ouest du Bénin. L’étude a été menée pendant la saison des pluies de juillet à novembre 2014 dans les établissements de santé locaux. Le gène *K13-propeller* a été amplifié dans les isolats de parasites par PCR imbriquée, et ensuite séquencé.

**Résultats :** Un total de 108 enfants a été recruté dans l’étude. L’efficacité des réactions d’amplification a été de 72 % (78/108). Le gène *K13-propeller* a été séquencé avec succès dans 78 isolats de *P. falciparum* ; tous étaient de type sauvage sans polymorphisme détectable.

**Conclusion :** l’absence de mutations dans le gène *K13* indique que les populations du parasite *P. falciparum* dans la zone d’étude sont encore pleinement sensibles aux artémisinines.

## Introduction

Artemisinin-based combination therapies are recommended by the World Health Organization (WHO) as first-line treatment for uncomplicated falciparum malaria in all areas in which malaria is endemic [32]. In 2004, as a result of high failure rates of treatment recorded with chloroquine and sulphadoxine-pyrimethamine (unpublished data from the National Malaria Control Programme), the Beninese National Malaria Control Programme implemented artemisinin-based combination therapy (ACT) as the first-line treatment for uncomplicated malaria. The artemether-lumefantrine combination was thus deployed throughout the country in health facilities.

Currently, the different ACTs in use remain highly effective for the treatment of malaria in Africa, as demonstrated both by rapid parasite clearance and low rates of recrudescence after therapy in clinical trials, as well as by the high rates of sensitivity of clinical isolates *ex vivo* [3, 16, 19, 27, 31, 35]. The first clinical cases of artemisinin resistance in western Cambodia were reported in 2008 [21], and *Plasmodium falciparum* with reduced *in vivo* susceptibility to artesunate was reported in 2009 [8, 9]. Emergence of resistance was subsequently reported in neighbouring regions [2, 11, 25]. These recent developments have grave implications for public health, since artemisinin derivatives are the mainstay of anti-malarial treatment worldwide. Hence the spread of ACT resistance could be catastrophic for malaria control and elimination efforts around the globe.

Despite the absence, thus far, of mutations associated with artemisinin resistance in *P. falciparum* isolates from different areas of sub-Saharan Africa [7, 20], previous experience with the spread of chloroquine and sulphadoxine-pyrimethamine resistant parasites from Asia to Africa [18, 33] demonstrates that the spread of drug resistance is likely, and that vigilant surveillance for resistant parasites is warranted. Recently, mutations in the propeller domain of the *K13* gene were identified as candidate molecular markers of artemisinin resistance associated with slow parasite clearance rates [1, 6, 17, 28]. These associations indicate that mutations in the *K13 propeller* (especially C580Y, R539T and Y439H) are important determinants of artemisinin resistance. These markers could therefore serve as a tool to monitor resistance to ACT. Although ACT remains highly efficacious for the treatment of falciparum malaria, and delayed parasite clearance after ACT has not been noted in Benin [15, 22], the molecular epidemiology of artemisinin resistance genotypes in Benin parasite populations is unknown. The aim of the study described here was to characterise the variability of the *K13* gene for the first time in Benin.

## Methods

### Study site

The study was conducted in Benin during the rainy season between July and November 2014, in two towns named Djougou, situated 450 km from Cotonou (the economic capital), and Cobly, 643 km from Cotonou. At the two sites, malaria transmission occurs from May to November during the rainy season. *P. falciparum* is the predominant parasite species transmitted by *Anopheles gambiae* (85%) and *An. arabiensis* (15%) [34]. The prevalence of *P. falciparum* infection in the general population was 19.1% in Djougou and 18% in Cobly (unpublished data).

### Patients, sample collection and laboratory procedures

*Plasmodium falciparum* isolates were obtained from children diagnosed with malaria who had lived in the area of the study sites for more than a period of 6 months and had not travelled during the previous month. Children visiting the health facilities in the study area, aged 6 months to 5 years, and who met the criteria below were enrolled in the study: (i) fever (axillary temperature ≥ 37.5 °C) or a history of fever within the past 48 h, (ii) *P. falciparum* mono-infection with parasite density ≥1,000 asexual forms per microlitre, identified by microscopy on blood smears; and (iii) written informed consent from parents. Venous blood from children fulfilling the above criteria was collected. Thick and thin blood smears were prepared, stained with 10% Giemsa and examined to determine *P. falciparum* density and to confirm mono-infection by *P. falciparum*. All thick blood smears were examined against 500 leucocytes. Parasite densities were recorded as the number of parasites/μL of blood, assuming an average leucocyte count of 8,000/μL of blood. All slides were read in the laboratories of the health centres, with external quality control performed on 10% of the negative slides and all positives in the reference Parasitology Laboratory of the *Centre National Hospitalo-Universitaire* in Cotonou. Evaluation of *K13*-propeller polymorphisms was performed using the same venous blood sample used for diagnostic analysis stored as spots on filter paper. All malaria-infected patients, based on microscopy results, were treated with standard doses of artemether/lumefantrine according to the national malaria treatment policy based on ACT.

### Analysis of *Plasmodium falciparum* isolates

Parasite DNA was extracted from filter paper using the Chelex method [26]. The propeller domain of the *K13* gene was amplified by nested PCR using the following primers: for the primary PCR (K13_PCR_F 5′-G GGAATCTGGTGGTAACAGC-3′ and K13_PCR_R 5′-C GGAGTGACCAAATCTGGGA-3′) and for the nested PCR (K13_N1_F 5′-GCCTTGTTGAAAGAAGCAGA-3′ and K13_N1_R 5′-GCCAAGCTGCCATTCATTTG-3′). The reaction volume and amplification programme used were reported previously [1]. Amplified products were bi-directionally sequenced by Sanger sequencing using BigDye^®^ v3.1 from ThermoFisher Scientific by Beckman Coulter Genomics. The sequence reactions were then run on an ABI3730xl following the manufacturer’s protocols. The propeller domain of the *K13* sequence data for single nucleotide polymorphisms (SNPs) was analysed using Geneious software (www.geneious.com). A cut-off of quality score HQ > 30% (the percentage of untrimmed bases that are high quality) was applied to all sequences. Sequences were assembled using the *de novo* assembly method and aligned to the reference *K13* annotated *Plasmodium falciparum* 3D7 (PF3D7-1343700). The polymorphism search was limited to inside CDS sequences. A search was performed for the mutations described in Asia and in the previous study [13, 14, 29].

*Ethical approval*: The study obtained the ethical approval of the National Ethics Committee for Health Research of Benin.

## Results

During the study period, a total of 225 potentially eligible patients were screened for participation in the study. Following application of inclusion criteria, a total of 108 participants were enrolled in the study. Children’s ages ranged from 6 months to 5 years (mean age: 31.6 ± 0.4 months). Parasite density ranged from 1,028 to 192,715 parasites/μL with a geometric mean density of 16,562 [9,909; 27,681]. Parasite DNA from the 108 *P. falciparum* isolates was analysed for *pfK13* genes. The efficiency of amplification reactions was 72.2% (78/108).

### Polymorphism of the *K13* propeller

The propeller domain of the *K13* gene was successfully sequenced in 78 *P. falciparum* isolates. After alignment with PF3D7-1343700, all the strains were found to be wild type having no polymorphism previously found in the *K13* gene.

## Discussion

In Benin, ACT was introduced as the first-line treatment for uncomplicated *P. falciparum* malaria in 2004. Although this treatment remains highly efficacious, it is important to monitor the potential presence of artemisinin-resistant *P. falciparum* parasite populations. The fact that only 72% of included samples were genotyped could be explained by the sensitivity of the PCR method used, because we did not analyse samples with low parasitaemia.

In this study, we did not find any mutation in the propeller part of the gene, as was the case in the Chatterie study in India [5]. Clinical artemisinin resistance is defined as a reduced parasite clearance rate, expressed as an increased parasite clearance half-life or a persistence of microscopically detectable parasites on the third day of ACT. The half-life parameter correlates strongly with results from the *in vitro* ring-stage survival assay. The absence of mutations in the *K13* gene of parasite strains isolated in Benin confirms the results of the therapeutic efficacy tests, conducted at the same study sites, where adequate clinical and parasitological response was 100% after PCR correction [22]. Thus, after 10 years of ACT use, no polymorphisms have appeared in the *K13* gene, suggesting that *P. falciparum* populations in the north-western part of Benin are still effectively susceptible to artemisinin. However, a larger sample size with extension into other parts of the country, including the south where drug pressure is higher [23], would allow us to draw better conclusions in this context. Artemisinin resistance, with delayed clearance of parasites after treatment with artemisinin monotherapy or artemisinin combination therapies (ACTs), is of great concern but has not yet been documented in sub-Saharan Africa, where speed of clearance of parasites after treatment with ACTs has generally been within the expected range [3, 10, 24, 31]. Small numbers of parasites with *K13*-propeller gene polymorphisms have been described in some countries in Africa, but importantly these were not the mutations previously associated with drug resistance in Southeast Asia [7, 20, 30]. The absence thus far of *K13* resistance-associated mutants from Southeast Asia in Africa is promising, but continuous surveillance for the emergence of resistance should be implemented to enable early detection. The use of molecular markers such as *K13* mutations is nowadays a cornerstone of malaria surveillance programmes, but potential differences between African and Asian *K13-*mutant parasites should be taken into account. The polymorphisms associated with artemisinin resistance in *P. falciparum* in Southeast Asia are not present in sub-Saharan Africa, but numerous *K13*-propeller coding polymorphisms have been documented in Africa [12–14, 24, 29]. Although their distributions do not support a widespread selective sweep for an artemisinin-resistant phenotype, the impact of these mutations on artemisinin susceptibility is unknown and will require further characterisation. Longitudinal studies conducted in Kenya [4, 20] showed that parasites from only one of 32 patients carried a mutation (at codon 578 (A578S)) in the propeller region of the *pfK13* gene, despite evidence of longer than normal parasite persistence in over 30% of children. To identify *K13* polymorphisms that affect artemisinin sensitivity in Africa, clinical trials should be supplemented with *in vitro* and molecular studies, providing additional data to strengthen confidence that observed mutations are associated with slowed parasite clearance.

## Conclusion

In this study, the absence of mutations in the *K13*-propeller gene suggests that artemisinin resistance is not a problem in Benin. However, similar studies from different parts of the country with a larger number of samples will be helpful to ascertain the emergence of artemisinin resistance, if any. In addition, routine monitoring and surveillance, as recommended by the WHO global plan for artemisinin resistance containment, should be continuously strengthened. Moreover, this study contributes to the ongoing surveillance of suspected artemisinin resistance parasites in Africa by providing baseline data on *K13*-propeller mutations in Benin.

## Conflict of interest

The authors declare no conflict of interest in relation with this paper.
